# To Our Readers

**Published:** 1892-10

**Authors:** 


### TO OUR READERS.

As will be seen, we present to our readers this month a London letter, which is full of interest and gives in a graphic style the news of that great metropolis. We have completed arrangements whereby we will be enabled also to publish a regular New York letter which will be fuller and more complete than those of the past.

We also offer this month some premiums, which will be found by turning to our advertising pages. It is our purpose to keep thoroughly abreast with the times, and from time to time, will add any new features which will be found advantageous to our readers.

---

Dr. Thos. F. Wood, of Wilmington, N. C., editor of the *North Carolina Medical Journal*, died suddenly on August 22nd, of aneurism of the aorta in its ascending portion. He was a man who enjoyed the respect and confidence of his colleagues, and up to his death did a very extensive practice. He was also Secretary of the State Board of Health, and a member of the Committee for the Revision of the Pharmacopœia. The profession has lost a brilliant light, and the public a benefactor.

---

The Mississippi Valley Medical Association will hold its eighteenth annual session at Cincinnati, Wednesday, Thursday and Friday, October 12th, 13th and 14th. The program is a valuable one, and covers every department in medicine.

---

SALOL IN CHOLERA.—The treatment for cholera proposed by Dr. Lœwenthal (a dose of two grams followed by hourly or half-hourly doses of one half to one gram of salol), after experimenting with it in the laboratory and on animals has been used on human beings with remarkable results. Dr. Gonzales, of Salvador, has used this treatment in fifty-three cases of cholera in one of the Phillipine Islands with only three deaths (and these were already in the last stages of the disease when they came to treatment). The mortality under other modes of treatment is about forty-five per cent.—*Ex.*

